# Biologics as novel therapeutics for the treatment of allergy: Challenges and opportunities

**DOI:** 10.3389/falgy.2022.1019255

**Published:** 2022-10-24

**Authors:** Amanda Atanasio, Jamie M. Orengo, Matthew A. Sleeman, Neil Stahl

**Affiliations:** ^1^ Immunology and Inflammation, Regeneron Pharmaceuticals, Inc., Tarrytown, NY, United States; ^2^ Research and Development, Regeneron Pharmaceuticals, Inc., Tarrytown, NY, United States

**Keywords:** allergy, biologics, type 2, immunotherapy, igE, IL-4/13, blocking igG antibodies

## Abstract

Over the last 4 decades there has been a significant global increase in the incidence and prevalence of IgE-mediated allergy. Although much progress has been made in the management of allergy *via* patient education, pharmacotherapy and immunomodulatory treatment regimens, significant unmet need remains. Advancements in our knowledge base surrounding the type 2 immune response, production of IgE and maintenance of immunological memory has led the field to explore targeted intervention of allergic pathways using monoclonal antibodies (mAbs). Intervention at various stages of the allergic cascade offers the opportunity to prevent initiation and/or maintenance of the type 2 immune response and effectively provide therapeutic benefit to patients. Furthermore, a better understanding of the protective mechanisms involved in allergen specific immunotherapy (AIT) has led us to appreciate the interplay of immunoglobulins in the allergic response, specifically the benefit in shifting the IgG:IgE ratio in favor of functionally relevant blocking IgG. Thus, treatments that lower IgE or boost IgG with the ability to outcompete IgE binding to allergen also present a favorable approach in the treatment of allergy. In this short review we discuss and highlight recent advances in the use of biologics to treat severe allergy, highlighting the key challenges but also the significant opportunities and advances to date.

## Introduction

Over the last 4 decades there has been a significant increase in the incidence and prevalence of allergy across the globe creating a significant burden on patients, healthcare providers and society. It was recently estimated that allergic rhinitis (AR) alone affects 10%–30% of the population worldwide, with rates as high as 50% in some countries (1, 2). Although first described in ancient Greece by Hippocrates (3) our modern understanding of allergy began in the late 18th and early 19th century. Seminal work by Prausnitz and Kustner in 1921 showed that transfer of a blood borne protein, later discovered to be immunoglobulin E (IgE) (4, 5) from a fish allergic individual to the skin of a non-allergic subject resulted in a hypersensitivity response upon exposure to fish extract at the site of transfer (6). Due to observations such as this and the discovery of cell types like mast cells and basophils (7) we now have built a good, but not complete, understanding of the key mechanisms, cellular players and inflammatory mediators that promote the allergic response.

The type 2 immune response plays a role in barrier immunity on mucosal surfaces and provides protection against large extracellular parasites. Type 2 immunity involves cooperation of the innate and adaptive immune system and is driven by a complex cytokine network. The response is characterized by production of epithelial cell-derived cytokines interleukin (IL)-25, thymic stromal lymphopoietin (TSLP), and IL-33 that are released at sites of initial allergen exposure as well as downstream production of IL-4, IL-13, IL-5 and IL-9. Subsequent differentiation of CD4^+^ T helper type 2 cells (Th2) results in recruitment of inflammatory effector cells (e.g., eosinophils, mast cell and basophils), goblet cell hyperplasia, mucus secretion and antibody class switch in favor of IgE production (8, 9). Acute hypersensitivity is an allergic reaction caused by allergen-induced crosslinking of IgE molecules bound to Fc-epsilon receptors (FcɛR) on the surface of mast cells and basophils. In a process termed “sensitization,” specific IgE is produced in response to allergen and binds FcɛRI on the surface of allergic effector cells. In a sensitized individual, subsequent exposure to the offending allergen may result in allergen binding to IgE and crosslinking of the IgE:FcɛRI complex, triggering degranulation and release of inflammatory mediators. This so-called early phase response occurs immediately after exposure and correlates with symptoms ranging from mild congestion, sneezing, and itching to more severe systemic reactions including urticaria, bronchoconstriction and potentially life-threatening anaphylaxis. Allergen uptake by antigen presenting cells further promotes activation of allergen-specific T cells and continued production of IgE. Together with infiltration of the mucosa by eosinophils, neutrophils, basophils and T cells, these events comprise the late phase response collectively resulting in sustained inflammation (10, 11).

Although progress has been made in the management of allergy *via* pharmacotherapeutics targeting symptom control (2), and the use of immunotherapy to potentially tolerize individuals to allergens (12, 13), significant unmet need remains. In this short review we discuss recent advances in the use of monoclonal antibody-based therapies to treat severe allergy (summarized in Table 1), and highlight the key challenges as well as significant opportunities.

**Table 1:** Biologics evaluated for the treatment of allergy.

Class of Therapy	Target	Therapeutic	Primary Mode of Action	Associated Studies
**Allergen Specific Monoclonal IgG**	**Fel d 1 (major cat allergen)**	**REGN1908-1909**	Binds allergen to prevent allergen engagement with IgE bound to FcR1 or CD23 thus inhibiting allergic effector cell activation and facilitated allergen presentation	Phase I: Single dose prevented acute allergic symptoms following NAC and reduced cat allergen SPT response (14–16)
				Phase II: Single dose prevented cat allergen induced reduction in FEV_1_ in cat allergic patients with mild asthma at d8 and up to d85 days after dose (17)
				Phase III: Field study ongoing in cat allergic participants (NCT04981717)
	**Bet v 1 (major birch allergen)**	**REGN5713-5714-5715**		Phase I: Single dose prevented acute allergic symptoms following NAC and reduced birch and alder SPT response; suppression of basophil responsiveness to birch, alder, hazel and apple also reported (18)
				Phase II/III: Studies ongoing in birch allergic participants (NCT05430919, NCT04709575)
**Type 2 Cytokines**	**IL4Ra**	**Dupilumab**	Binds to the IL4Ra receptor and inhibits signaling of both IL-4 and IL-13	Post-hoc and observational studies report reduction in AR-associated nasal and ocular symptoms in patients treated with dupilumab for atopic dermatitis and/or asthma (19–22)
				Phase II: Addition of dupilumab to 16 weeks of TG SCIT improved tolerability of SCIT up-titration but did not reduce acute allergic symptoms following NAC as compared to SCIT alone (23, 24)
				Phase II: Monotherapy study and adjunct to peanut OIT study in peanut allergic participants ongoing (NCT03793608, NCT03682770)
**Epithelial Derived Cytokines**	**TSLP**	**Tezepelumab**	Binds TSLP preventing receptor interaction and signaling	Phase I/II: Addition of tezepelumab to 52 weeks of cat SCIT decreased allergic symptoms following NAC as compared to cat SCIT alone during treatment; effect not sustained one year after completion of therapy (25)
	**IL-33**	**Etokimab**	Binds IL-33 preventing receptor interaction and signaling	Phase II: Single dose increased the tolerated threshold allergen amount in comparison to placebo in peanut allergic adults at d15 and d45 (26)
**Anti-IgE**	**IgE**	**Omalizumab**	Binds to the Fc region of IgE to prevent/disrupt IgE engagement with FcR1 and CD23	Post-hoc and observational studies report reduction in AR-associated nasal and ocular symptoms and food allergic symptoms in patients treated with omalizumab for allergic asthma (27–29)
				Phase I/II: Addition of omalizumab to SCIT for various aeroallergens reduced AR and asthma symptoms as compared to SCIT alone (30, 31)
				Phase I/II: Addition of omalizumab to OIT for peanut or multi-food allergy facilitated desensitization and improved efficacy and safety as compared to OIT alone (31–33)
				Phase III: Omalizumab as a monotherapy and as adjunct therapy to multi-allergen OIT in food allergic participants (NCT03881696)
	**IgE**	**Ligelizumab**	Binds to the Fc region of IgE to prevent/disrupt IgE engagement with FcR1 and CD23	Phase III: Efficacy of ligelizumab monotherapy in peanut allergic participants ongoing (NCT04984876)
	**Membrane IgE**	**Quilizumab**	Binds to the extracellular membrane-proximal domain of IgE bound to B cells to induce apoptosis; promotes ADCC *via* interaction with FcRa on NK cells	Phase I/II: Quilizumab for the treatment of AR or allergic asthma demonstrated reduced total and allergen specific IgE but only modest improvement in allergen-induced asthmatic airway response that did not repeat in additional studies (34)

FEV_1_, forced expiratory volume in one second; ADCC, antibody dependent cell-mediated cytotoxicity; NK, natural killer.

### Allergen specific immunotherapy

AIT is a treatment option for type 1 hypersensitivity when first-line pharmacotherapies prove insufficient. AIT involves administration of increasing doses of allergen over months to years with the goal of inducing tolerance. Efficacy has been established for aeroallergens, bee venom and most recently, peanut allergy (35, 36). While AIT has the potential to be disease modifying, the use of heterogeneous allergen mixtures can lead to variable results and high rates of reactions ranging from mild to severe and life-threatening anaphylaxis. Mild side effects include rhinitis or <20% reduction in peak expiratory flow (PEF) with reactions such as urticaria, angio-oedema or >20% reduction in PEF classified as severe. Although rare, anaphylactic shock is also reported and risk factors for any such adverse events are largely unknown (37). Furthermore, treatment length may require 3–5 years to achieve clinical benefit but poor patient compliance and often waning responses following completion of therapy means the benefits are variable and limiting. Collectively there is great interest in developing approaches that decrease risk, reduce treatment length, and sustain desensitization (promote tolerization). The use of biologics that mimic protective mechanisms of AIT or target major drivers of the allergic response administered alone or in conjunction with AIT presents such opportunity for safer and more effective approaches.

Multiple hypotheses exist to explain the protective mechanisms of AIT, as it is thought to modify both the cellular (reduced Th2 phenotype and induction of T-regulatory cells) and humoral response (12). In a series of classical experiments, a “transferable protecting substance” later referred to as “blocking IgG” was identified in the serum of patients undergoing AIT (38–40). Further studies confirmed induction of allergen-specific IgG and IgA antibody titers after AIT initiation (41–43), but revealed this quantitative measurement alone is not a good surrogate of clinical efficacy. Rather functional blocking activity of these antibodies, namely their ability to compete with IgE for allergen binding, was shown to correlate with clinical symptoms (44, 45). Such data supports the notion that just a few, high-quality, functionally important anti-allergen antibodies can be effective in allergic disease and the diverse antibody response that occurs in most patients likely explains the unpredictable and variable clinical response seen with AIT. Overall, these observations laid the framework for the concept that identifying potent allergen specific blocking antibodies could provide protection from allergen induced hypersensitivity reactions (46, 47).

## Passive administration of allergen blocking IgG

**Figure 1:**
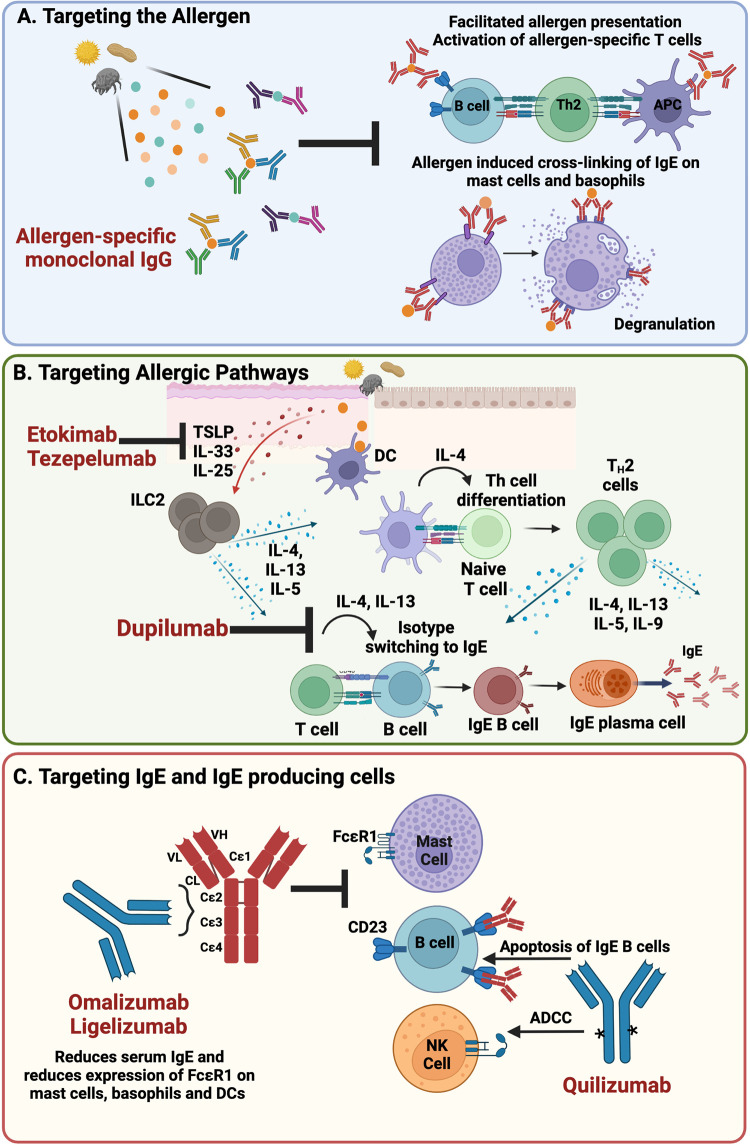
Targeted interventions for the treatment of IgE-mediated allergy (**A**). Passive administration of blocking IgG that binds allergen and inhibits allergen:IgE engagement on the surface of allergic effector cells prevents initiation of the allergic response and facilitated allergen presentation (**B**). Blockade of the epithelial-derived cytokines, TSLP and IL-33 and type 2 cytokines IL-4 and IL-13, present opportunities to inhibit initiation and maintenance of the allergic response (**C**). Directly targeting IgE and IgE-producing cells lowers the levels of IgE in circulation and decreases expression of FcɛR1, the high affinity IgE receptor, on allergic effector cells thus blunting the allergic response (Made with Biorender.com); DC, dendritic cell; NK, natural killer; ADCC, antibody dependent cell-mediated cytotoxicity.

Passive immunotherapy with antibodies against antigens is a treatment approach that has long been utilized in various settings. Its utility has extended to targeted treatment and prevention of viral infection using purified monoclonal antibodies (mAbs) with the advent of Palivizumab, a single mAb treatment for respiratory syncytial virus (48) and, more recently the use of mAb cocktails in the fight against deadly viral outbreaks of Ebola and SARS-CoV-2 (49–52). Although a well-established approach, passive administration of allergen-specific mAbs presents a novel application of passive immunotherapy with the potential to provide a rapid and reliable treatment option for specific allergy (Figure 1A).

The concept of passive administration of allergen specific blocking mAbs for the treatment of allergy was first validated in a small proof of mechanism study in cat allergic individuals and extended to a study in birch allergic individuals shortly thereafter (14, 18). Specifically, REGN1908-1909, a mAb cocktail targeting two non-overlapping epitopes on the major cat allergen, Fel d 1, and REGN5713-5714-5715, consisting of three mAbs targeting distinct epitopes of Bet v 1, the major birch allergen, were developed using the Velocimmune antibody platform (14, 53–55). Antibodies were selected based on binding affinity and IgE blocking potency in a series of preclinical assays. In each clinical study, a single subcutaneous administration of the allergen specific neutralizing antibody cocktails prevented acute allergic symptoms following nasal allergen challenge (NAC) as measured by a significant reduction in total nasal symptom score (TNSS) relative to baseline as compared to placebo control. Furthermore, significant reductions in allergen-specific skin prick test (SPT) were observed which is consistent with the proposed mechanism of action of the approach: antibody-allergen engagement to prevent interaction with allergen specific IgE on the surface of effector cells. In addition, allergen neutralizing capacity was observed in the peripheral blood (18) as well as the local target organ (nasal fluid) (15). The cocktail of Fel d 1 mAbs further demonstrated a rapid and durable reduction in cat allergen induced bronchoconstriction in cat allergic patients with mild asthma, preventing early asthma reactions (defined as a ≥20% decline in forced expiratory volume over one second) as early as one week after treatment and up to three months, in addition to improved lung function and increased amount of cat allergen that patients could tolerate (17). Given these promising early results, the Fel d 1 mAb cocktail and the Bet v 1 mAb cocktail are currently being investigated in larger phase III studies (NCT04981717, NCT04709575).

Notably, vaccines for the treatment of allergy also rely largely on the induction of blocking IgG, as do emerging AIT approaches aiming to achieve protective effects whilst limiting the risk of IgE-mediated side effects through the use of allergen components or allergoids modified to reduce allergenicity yet maintain an ability to mount humoral responses towards functional blocking IgG (56–60). Although passive administration of anti-allergen blocking antibodies is not expected to result in immunological memory as AIT or vaccinology have been reported to achieve, it presents an efficient approach to obtaining high titers of quality allergen-specific blocking IgG with rapid onset of action. It further avoids the need for repeated administrations of allergen or allergen derivatives that can induce unwanted allergic symptoms, thus broadening the potential patient population to include asthmatics contraindicated to receive AIT and is not reliant on the patient's immune system to achieve high antibody titers of functional blocking IgG.

Fundamental to this is approach is a robust understanding of the structural basis of allergens and allergen antibody interactions. Multiple epitopes of an allergen likely need to be blocked to achieve robust inhibition of allergic effector cell activation. Although it has been shown that maximal coverage of an allergen is not necessary to achieve blockade of the IgE mediated response (14, 55) defined, immunodominant allergens with a finite number of epitopes are favorable as more complex allergen components/epitopes may prove difficult to target. Polyallergy is an additional challenge to overcome, as individuals with a single allergy are rare. However, it is not unreasonable to speculate that targeting a patient's major driver of allergy may lower their overall allergic threshold, thereby providing symptomatic relief (61, 62). Finally, although PK/PD studies demonstrate that efficacious mean target concentrations of anti-allergen mAbs are maintained for 8–12 weeks suggesting potential for infrequent dosing (16), treatment regimen and length for this approach remains to be determined.

## Targeting type 2 cytokines

Other approaches target cytokines involved in the development and/or maintenance of allergy. Of particular interest are type 2 cytokines, such as IL-4, IL-13 and IL-5 and the epithelial cell-derived cytokines IL-25, TSLP, and IL-33 that are released at sites of initial allergen exposure (Figure 1B).

The cytokines IL-4 and IL-13 play prominent roles in both the induction and effector phases of the type 2 immune response driving Th2 polarization, eosinophil and T-cell trafficking to tissue, activation of B cells and induction of B cell class switching to IgE, all of which are fundamental features of allergic disease (8, 63). Dupilumab, a fully human monoclonal antibody that binds to IL-4 receptor alpha (IL-4R*α*) inhibits signaling of both IL-4 and IL-13 and is approved for the treatment of atopic dermatitis, asthma, chronic sinusitis with nasal polyps and eosinophilic esophagitis (64). Across multiple atopic diseases, dupilumab has been shown to suppress type 2 inflammatory biomarkers, including total and allergen specific IgE (19), and reduce AR symptoms in patients with perennial AR and comorbid asthma or AD (20–22). To this end, dupilumab was evaluated in a 16-week treatment course as a monotherapy or as an adjunct to subcutaneous immunotherapy (SCIT) for the treatment of timothy grass (TG) pollen allergy. While dupilumab plus TG SCIT did not reduce TNSS following TG NAC compared with SCIT alone, the combination did improve tolerability of SCIT up-titration as evidenced by fewer treatment discontinuations due to adverse events, reduced need for epinephrine as rescue medication and a higher proportion of patients achieving the target SCIT maintenance dose compared to SCIT alone (23, 24). Biomarker evaluation revealed that dupilumab in combination with SCIT significantly reduced sIgE levels and increased the log sIgG4/sIgE and sIgG/sIgE ratios compared with SCIT alone presenting a possible mechanism by which dupilumab may improve the tolerability of SCIT. These key exploratory and biomarker findings suggest further study may be warranted. It is possible that a longer treatment course may better address the utility of IL-4/IL-13 blockade in this setting, as the reduction in total IgE is believed to be gradual post dupilumab treatment, with 70%–75% reduction achieved at 52 weeks (19). It is also possible that evaluation of efficacy using an AR-induced NAC model, which primarily evokes early allergic responses in sensitized individuals, further limits the findings. Perhaps evaluating the approach over a pollen season as done in previous studies reporting positive outcomes of dupilumab in AR (20–22) may better address its utility for allergy.

Tezepelumab, a TSLP neutralizing monoclonal antibody approved for the treatment of asthma, was tested in a similar setting with a focus on tolerance induction as an add on to cat immunotherapy. In this randomized, double blind, placebo controlled 4 arm study (cat-SCIT alone, cat-SCIT with tezepelumab, tezepelumab alone or placebo), patients received 52 weeks of treatment followed by 52 weeks of observation off therapy. As tolerance induction was the goal, the primary endpoint of the study was defined as a significant reduction in TNSS in response to NAC in the cat-SCIT with tezepelumab group as compared to cat-SCIT alone at week 104 following completion of 52 weeks on therapy and 52 weeks with no treatment. Although those who received cat SCIT with tezepelumab did achieve a significant reduction in TNSS in response to NAC as compared to cat SCIT alone during treatment (week 25 and 52), the effect was not sustained following completion of therapy (week 104) and thus the study did not meet its primary endpoint (25).

The epithelial cytokine IL-33 is another emerging therapeutic target for allergic disease. In an exploratory Ph2a clinical trial for peanut allergy, treatment with a single dose of etokimab, a humanized monoclonal antibody specific for IL-33, increased the tolerated threshold allergen dose (73% at day 15% and 57% at day 45) in comparison to placebo (0% at day 15 and 45). Reported differences in select biomarkers further suggest etokimab may interfere with downstream allergic pathways (IgE production, T cell activation) (26). While promising, the small sample size (*n* = 15 active, *n* = 5 placebo), short study duration, high dropout rate and an imbalance in baseline characteristics warrants a more comprehensive interrogation to determine a role for IL-33 in food allergy. Itepekimab is an additional fully human IL-33 neutralizing monoclonal antibody under clinical development. Although not yet evaluated for the treatment of allergy, it was shown to affect a lower incidence of loss of asthma control vs. placebo and improved lung function in a ph2 study for moderate to severe asthma (65, 66) as well as potential benefit for COPD (67) patients with ph3 studies ongoing.

We have more to learn about the role of type 2 and epithelial cytokines as the drivers of established disease especially in the context of AIT. These studies have only begun to scratch the surface of our understanding with several additional clinical trials actively ongoing, evaluating various treatment regimens as well as interventions at the point of prevention in high-risk individuals (AD patients/genetic risk factors).

## Anti-IgE therapy

IgE is the immunoglobulin subclass that plays the central role in acute allergic hypersensitivity therefore presenting as an overt target for the treatment of allergy (Figure 1C). Omalizumab is a first in class humanized monoclonal antibody specific for IgE, approved for the treatment of allergic asthma in 2003 and subsequently for the treatment of chronic spontaneous urticaria (CSU) and nasal polyps. Omalizumab binds to the Fc-domain of IgE and prevents IgE binding to the high affinity IgE receptor, FcɛRI, as well as the low affinity IgE receptor FcɛRII (CD23) (68). Free IgE levels drop rapidly within 1hr of a patient being dosed with omalizumab, with up to 99% reduction observed thus reducing the availability of free IgE to bind FcɛRI. Expression of FcɛR1 is also decreased on the surface of basophils, mast cells and dendritic cells which combined with the drop in free IgE results in decreased allergic effector cell activation and sensitivity (69). Early trials demonstrated that omalizumab could significantly reduce nasal and ocular symptoms of seasonal AR, prevent the need for rescue medications such as antihistamines (27) and reduce food allergic symptoms in patients with allergic asthma (28). Similarly, omalizumab also reduced asthma symptoms in cat allergen sensitized subjects (29). Whilst these studies were positive, the level of clinical benefit was overall modest. Although capable of reducing free IgE in circulation, omalizumab's ability to displace pre-bound cell surface IgE on mast cells is limited due to the slow dissociation rate and higher affinity interaction of IgE and FcɛRI (70, 71) than omalizumab for the Fc domain of IgE. Interestingly new molecules are currently being developed to better displace IgE from its receptors (72–74) but it is not clear whether these will be taken forward in allergy or the more traditional route of severe asthma.

More recently, studies have focused on using omalizumab in combination with immunotherapy to enhance efficacy and safety of such tolerizing approaches. AIT in combination with omalizumab for the treatment of AR was first evaluated in 2002 in a randomized double blind placebo controlled (DBPC) study of 221 children with birch and/or grass pollen allergy. Omalizumab in combination with SCIT reduced symptoms by 48% over 2 pollen seasons when compared with SCIT alone (30). Additional observations in the omalizumab + SCIT group included reduced symptom severity, reduced need for rescue medication and fewer days with allergy symptoms as compared to SCIT alone. The combination effect in this study was irrespective of allergen; birch or grass, highlighting omalizumab as an antigen independent approach with potential to broadly benefit subjects with polyallergy. Additional clinical trials and case studies have explored aeroallergen AIT in various settings such as pretreatment to AIT followed by rush updosing, as well as in high-risk asthmatic populations contraindicated to undergo AIT (31). Results from these studies are largely positive however omalizumab is yet to seek approval for such indications.

Clinical trials evaluating Omalizumab as an add on to oral immunotherapy (OIT) for food allergy have also been conducted with a primary focus on milk, peanut and more recently multifood OIT (31). Early studies in individuals with milk allergy demonstrated improved tolerability during rapid dose escalation OIT with the addition of omalizumab; however final effectiveness as determined by oral food challenge (OFC) was not significantly different between OIT and OIT + omalizumab treatment groups (32). The first DBPC randomized study investigating the efficacy of omalizumab in conjunction with peanut OIT showed that omalizumab facilitated oral desensitization in peanut allergic subjects (33). In this study, omalizumab was dosed 12 weeks before initiating rapid oral desensitization. At the start of the rapid desensitization protocol, 23/27 subjects in the omalizumab pre-treatment group completed 250 mg of desensitization in contrast to the placebo group where only 1/8 tolerated that amount. Likewise, 21 of those omalizumab treated subjects were able to tolerate a weekly increase of peanut up to 2000 mg over 20 weeks, with only a single subject in placebo achieving this level of desensitization. Upon withdrawal of omalizumab 75% of subjects in the treatment population could tolerate 2000 mg of peanut 6 weeks after the last dose, whereas only 12.5% of placebo subjects could. Despite promising efficacy, it's worth noting that OIT associated side effects were still reported in such studies, namely development of eosinophilic esophagitis, suggesting that strategies targeting IgE may not fully shut down the allergic response. Nonetheless, Phase III clinical trials are underway to further evaluate the utility of omalizumab as an adjunct therapy to OIT as well as to revisit efficacy as a monotherapy in food allergy.

The next generation anti-IgE molecule, ligelizumab, a humanized monoclonal IgG1 mAb recognizing an IgE-epitope distinct from that of omalizumab, is also in clinical development for the treatment of allergic disease (Figure 1C). Ligelizumab also binds the Fc portion of IgE but shares significantly more overlap with the binding epitope of FcɛR1 on IgE than omalizumab, suggestive of a potential therapeutic advantage in FcɛRI-driven allergic disease. An additional distinction is ligelizumab is less potent at interrupting IgE:FcɛRIIb (CD23) interactions than omalizumab (34, 75). Of note, ligelizumab initially showed significantly better symptom control over omalizumab in a phase2b clinical study for CSU however these results were not replicated in a larger Phase 3 setting. In addition, while omalizumab is approved for the treatment of asthma, ligelizumab did not significantly improve asthma control or exacerbation rates compared to omalizumab or placebo (76). Together findings from these two studies suggest that these mechanistic/binding differences are important in different disease settings. Ligelizumab is currently under evaluation in a Ph3 multicenter study as a monotherapy for peanut allergy. Although presenting a similar protective mechanism as omalizumab it is of interest to know if ligelizumab will provide benefit.

### Targeting the source of serological memory

While anti-IgE therapy shows promise as a potential treatment option for patients with severe food allergies, identifying and targeting the source of IgE also offers promise. Clinical observations support the concept that such a source exists that maintains levels of circulating allergen specific IgE. For example, allergy can be transferred during a bone marrow transplant and persists long term (77, 78). It is also known that serum IgE is maintained in atopic patients in the absence of allergen (79). Lastly, serum IgE is reduced, but not abolished in patients treated with the above-mentioned approaches that target IgE class switching or IgE switched cells (80). For example, quilizumab, a monoclonal antibody that targets membrane IgE and depletes IgE switched cells (Figure 1C) has shown only a modest percent reduction in serum IgE (81). Likewise, dupilumab, a monoclonal antibody that binds to IL-4R*α*, inhibits IL-4 and IL-13 signalling and inhibits class switching to IgE shows approximately a 70 percent reduction in IgE after one year (19). These findings further support the notion that the source of IgE is not impacted by these approaches. A recent study demonstrated that IgE bone marrow plasma cells can maintain IgE serological memory, are long lived, and are driven from sequential class switching of IgG1 B-cells (82). This finding provides evidence that there are two sources of memory with respect to IgE: a cellular source, the IgG1 memory B-cell and a serological source, the IgE + PC and ultimately suggests that interventions to completely wipe out IgE from circulation could require a two-pronged approach.

## Conclusion

Targeted intervention in the prevention, initiation, or maintenance of the type 2 immune response holds the potential to effectively provide therapeutic benefit to patients. Understanding the interplay of immunoglobulins in the allergic response and effectively shifting the IgG:IgE ratio in favor of functionally relevant blocking IgG with the ability to outcompete IgE binding to allergen presents one favorable approach to provide protection. To treat allergy more broadly, directly targeting IgE or allergic pathways and cytokines involved in its production and maintenance offers promise. Overall, these studies highlight key mechanistic similarities and differences driving the allergic response and collectively emphasize the need for more studies in this space.
